# Conformational Flexibility in the Immunoglobulin-Like Domain of the Hepatitis C Virus Glycoprotein E2

**DOI:** 10.1128/mBio.00382-17

**Published:** 2017-05-16

**Authors:** Ieva Vasiliauskaite, Ania Owsianka, Patrick England, Abdul Ghafoor Khan, Sarah Cole, Dorothea Bankwitz, Steven K. H. Foung, Thomas Pietschmann, Joseph Marcotrigiano, Felix A. Rey, Arvind H. Patel, Thomas Krey

**Affiliations:** a Unité de Virologie Structurale, Department Virologie, Institut Pasteur, Paris, France; b CNRS UMR 3569, Paris, France; c MRC-University of Glasgow Centre for Virus Research, Glasgow, United Kingdom; d Plate-Forme de Biophysique Moléculaire, Institut Pasteur, Paris, France; e CNRS UMR 3528, Paris, France; f Center for Advanced Biotechnology and Medicine, Department of Chemistry and Chemical Biology, Rutgers University, Piscataway, New Jersey, USA; g Institute for Experimental Virology, Centre for Experimental and Clinical Infection Research, Twincore, Hannover, Germany; h Department of Pathology, Stanford University School of Medicine, Stanford, California, USA; i Institute of Virology, Hannover Medical School, Hannover, Germany; j German Center for Infection Research, Hannover-Braunschweig Site, Germany; Icahn School of Medicine at Mount Sinai

**Keywords:** CD81 binding site, hepatitis C virus, Ig-like domain, conformational flexibility, glycoprotein E2, monoclonal antibodies, vaccine design

## Abstract

The hepatitis C virus (HCV) glycoprotein E2 is the major target of neutralizing antibodies and is therefore highly relevant for vaccine design. Its structure features a central immunoglobulin (Ig)-like β-sandwich that contributes to the binding site for the cellular receptor CD81. We show that a synthetic peptide corresponding to a β-strand of this Ig-like domain forms an α-helix in complex with the anti-E2 antibody DAO5, demonstrating an inside-out flip of hydrophobic residues and a secondary structure change in the composite CD81 binding site. A detailed interaction analysis of DAO5 and cross-competing neutralizing antibodies with soluble E2 revealed that the Ig-like domain is trapped by different antibodies in at least two distinct conformations. DAO5 specifically captures retrovirus particles bearing HCV glycoproteins (HCVpp) and infectious cell culture-derived HCV particles (HCVcc). Infection of cells by DAO5-captured HCVpp can be blocked by a cross-competing neutralizing antibody, indicating that a single virus particle simultaneously displays E2 molecules in more than one conformation on its surface. Such conformational plasticity of the HCV E2 receptor binding site has important implications for immunogen design.

## IMPORTANCE

Recent advances in the treatment of hepatitis C virus (HCV) infection with direct-acting antiviral drugs have enabled the control of this major human pathogen. However, due to their high costs and limited accessibility in combination with the lack of awareness of the mostly asymptomatic infection, there is an unchanged urgent need for an effective vaccine. The viral glycoprotein E2 contains regions that are crucial for virus entry into the host cell, and antibodies that bind to these regions can neutralize infection. One of the major targets of neutralizing antibodies is the central immunoglobulin (Ig)-like domain within E2. We show here that this Ig-like domain is conformationally flexible at the surface of infectious HCV particles and pseudoparticles. Our study provides novel insights into the interactions of HCV E2 with the humoral immune system that should aid future vaccine development.

## INTRODUCTION

Hepatitis C virus (HCV) has infected an estimated 170 million people worldwide, and the majority of infected individuals develop chronic infection that leads to progressive liver disease (1). The recently approved combination therapies involving direct-acting antivirals have impressive cure rates (≥99%) (2), but their high costs limit accessibility, and so there is still an urgent medical need to develop a preventative HCV vaccine.

Most neutralizing antibodies (nAbs) identified to date target the viral envelope glycoprotein E2 (3), which interacts with the cellular receptors CD81 (4) and scavenger receptor BI (SR-BI) (5). E2 contains several hypervariable regions (6, 7) that can be deleted without affecting the overall glycoprotein conformation (8). Crystallization of E2 was a difficult endeavor, and crystal structures were reported only recently, via use of truncated versions of the E2 ectodomain (cE2), which lack some of these hypervariable regions and the C terminus, in complex with anti-E2 antibody fragments (9, 10). These difficulties suggested that HCV E2 is more flexible than glycoproteins of other viruses that have been successfully crystallized on their own. Further evidence for the flexibility of the HCV glycoproteins comes from the observations that the oligomeric status as well as the disulfide connectivity of the HCV glycoproteins fluctuate during the HCV replication cycle (11, 12).

**FIG 1:**
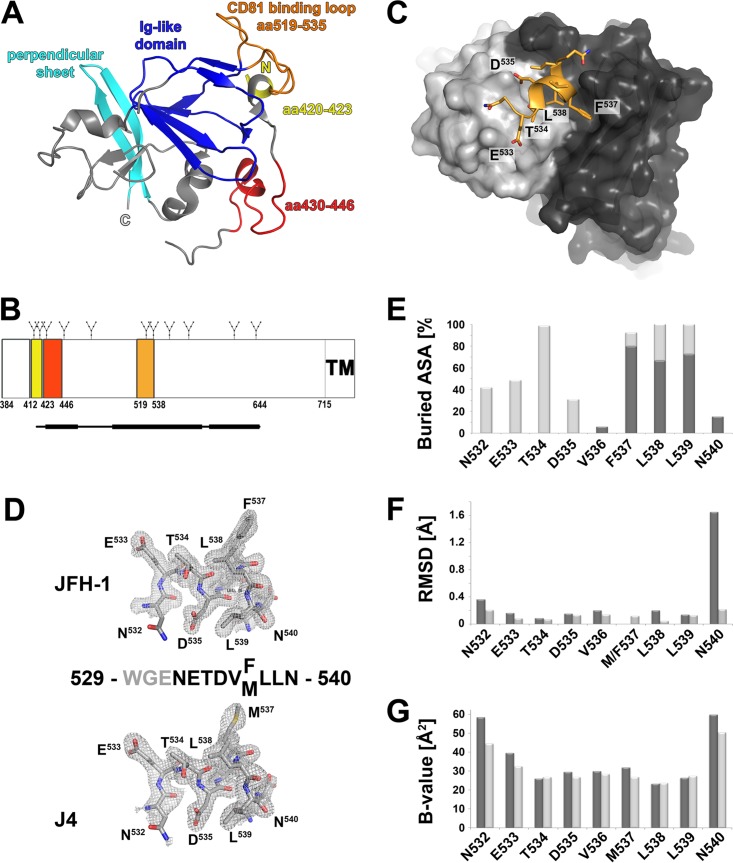
Crystal structure of DAO5 in complex with its peptide epitope. (A) Cartoon representation of cE2 (PDB 4WMF). The N and C termini are indicated; the Ig-like domain and the perpendicular sheet are highlighted in blue and cyan, respectively. The three segments contributing to the CD81 binding site are colored as described for panel B. (B) Linear diagram of the E2 glycoprotein. N-linked glycosylation sites are indicated above the diagram, and the three segments contributing to the composite CD81 binding site are shown as colored boxes and labeled with their amino acid residue numbers. A bar corresponding to the crystallized cE2 construct described in reference 10 is shown below the diagram, with the thinner line indicating disordered regions. (C) View of the DAO5 paratope in complex with the JFH-1 peptide (aa 529 to 540). The molecular surfaces of the light chain and heavy chain are colored light gray and dark gray, respectively, and the peptide is shown as a cartoon, displaying the side chains as sticks and colored according to atom type (orange, red, and blue for carbon, oxygen, and nitrogen, respectively). The α-helix formed by the C-terminal end of the peptide contacts the heavy chain CDR loops, while its N-terminal extended region (N532-T534) contacts the CDR loops of the light chain. (D) Electron density of the composite omit map contoured at 1σ around the peptide corresponding to HCV E2 strain JFH-1 (upper panel) or J4 (lower panel). The amino acid sequences of the peptides are displayed, with gray residues indicating disordered regions. (E) Percentages of accessible surface area (ASA) buried in the complex, calculated using PISA (51) and represented per residue as stacked columns for heavy (dark gray) and light (light gray) chains. (F) RMSD upon superposition of the two peptides, created using Chimera (52) and including all atoms (dark gray) or main chain atoms only (light gray) in the calculation and represented per residue. (G) Average temperature factors of the peptides plotted per residue for complexes with JFH-1 (light gray) and J4 (dark gray).

The two available cE2 structures revealed a central immunoglobulin (Ig)-like domain that is completed by random coil, small helices, and an additional β-sheet perpendicular to the Ig β-sandwich (Fig. 1A). The exposed C′-E loop of this β-sandwich, also called CD81 binding loop, comprises amino acid (aa) residues 519 to 535 (the amino acid numbering used here corresponds to the polyprotein of the HCV genotype 1a strain H77) and constitutes, together with two segments that are distant in the primary structure (aa 412 to 423 and aa 428 to 446), the composite CD81 binding site (10, 13, 14) (Fig. 1A and B). The CD81 binding loop is stabilized by binding of a Fab fragment in one of the two E2 structures (10) with the side chain of residues F^537^ and L^539^ located on β-strand E buried in the hydrophobic core of the Ig-like domain. However, in the second E2 structure, residues 524 to 535 are disordered and the side chain of F^537^ is solvent exposed (9), indicating that β-strand E is partially unfolded and the Ig-like domain can adopt more than one conformation in the absence of a stabilizing Fab fragment. The fact that the disulfide connectivity in the two reported cE2 structures differs in one disulfide bond (9, 10) further highlights the extensive conformational flexibility of HCV E2, which in many respects behaves differently than other viral glycoproteins by not adopting a single, rigid conformation.

Further insights into the structural organization of neutralizing epitopes within E2 came from cocrystallization of Fab fragments derived from nAbs in complex with their respective epitope peptides comprising E2 residues 412 to 423 or 430 to 446 (15–20). More recent structural studies using different antibodies in complex with similar epitope peptides revealed that these epitopes are either conformationally flexible (residues 412 to 423 [21–23]) or at least adopt two discrete conformations (residues 430 to 446 [24]). Moreover, electron microscopy analysis illustrated the flexibility of aa 412 to 423 as a neutralizing Fab targeting this segment bound soluble E2 from a variety of different angles of approach (25).

Here, we provide further evidence that the receptor binding part of the Ig-like β-sandwich within E2 can adopt distinct conformations at the surface of infectious particles (both virus particles bearing HCV glycoproteins [HCVpp] and infectious cell culture-derived HCV particles [HCVcc]), exposing residues that are part of the hydrophobic core and changing the secondary structure of one β-strand. We used two monoclonal antibodies (MAbs) that share contact residues but recognize distinct conformations of the Ig-like domain to detect the simultaneous presence of distinct protein conformations at the surface of a single HCVpp. Our results demonstrate that the previously described structural flexibility within HCV E2 extends beyond the composite CD81 binding site and also that the Ig-like domain undergoes conformational rearrangements. This report therefore provides novel structural insights into the conformational flexibility of receptor binding sites present in viral glycoproteins in general, and in particular of HCV E2, with implications for vaccine design.

## RESULTS AND DISCUSSION

### Crystallization of antibody fragment-peptide complexes and structure determination.

We generated and characterized anti-E2 MAb DAO5, which specifically recognizes a linear epitope comprising part of the CD81 binding loop and the E strand of the central β-sandwich within HCV E2 (see Text S1 and Fig. S1 in the supplemental material). The linear character of the DAO5 epitope makes it a useful tool for structural analysis of this functionally important region.

10.1128/mBio.00382-17.1 TEXT S1  Supplemental results, methods, and references. Download TEXT S1, DOCX file, 0.05 MB. Copyright © 2017 Vasiliauskaite et al. 2017 Vasiliauskaite et al. This content is distributed under the terms of the Creative Commons Attribution 4.0 International license.

10.1128/mBio.00382-17.2 FIG S1  Functional characterization of MAb DAO5. (A) Peptides corresponding to the sequence of the MAb DAO5 epitope competed for its binding to E2 in an enzyme-linked immunosorbent assay (ELISA). Lectin of *Galanthus nivalis* (GNA)-captured full-length E1E2 (genotype 2a JFH1), expressed in HEK cells, was probed in an ELISA with DAO5 in the presence of peptides spanning its epitope. A peptide corresponding to the MAb AP33 epitope (aa 411 to 424) was included as a negative control. Peptide sequences are shown, with the DAO5 epitope in boldface. (B) Reactivity of MAb DAO5 to E2 carrying an alanine substitution of conserved residues W529 and D535. Wild-type (WT) and mutant full-length E1E2 was expressed in HEK cells and captured on GNA-coated microtiter plates. The reactivities of serial dilutions of DAO5 with E2_wt_ (●), E2_W529A_ (■), and E2_D535A_ (▲) were tested alongside control MAbs AP33 and HC-1. (C) Neutralization of HCVpp and HCVcc. Genotype 2a JFH1 HCVpp or HCVcc were incubated for 1 h with an excess (100 µg/ml) of DAO5 or control antibodies prior to infecting Huh7 cells. Fab fragments were tested alongside whole MAbs in the HCVcc experiment. Infectivity levels in the presence of antibody, determined at 72 h postinfection, are presented as the percent infectivity in the absence of antibody. Values shown are the means and standard deviations of two independent experiments. Download FIG S1, TIF file, 0.7 MB. Copyright © 2017 Vasiliauskaite et al. 2017 Vasiliauskaite et al. This content is distributed under the terms of the Creative Commons Attribution 4.0 International license.

We expressed a Fab and a single-chain Fv (scFv) fragment of MAb DAO5 in *Drosophila melanogaster* S2 cells, as described elsewhere (26, 27). Diffraction-quality crystals of these antibody fragments were obtained in complex with peptides spanning E2 residues 529 to 540 of strains J4 (genotype 1b) and JFH-1 (genotype 2a) (see Text S1). Three independent structures were determined by using the molecular replacement method with the previously determined crystal structure of the unliganded scFv fragments as the search model (scFv-J4 peptide, scFv-JFH1 peptide, and Fab-J4 peptide). The final electron density maps revealed unambiguous density, allowing us to build independent atomic models of the peptides (Fig. 1C and D). Superposition of peptide residues 532 to 540 from Fab-J4 and scFv-J4 peptide complexes confirmed an identical peptide conformation with a root mean square deviation (RMSD) of 0.136 Å, calculated over the backbone atoms (Fig. 1F), which together with the unrelated crystal packing interfaces for the Fab and scFv complexes indicated that our crystal structures reflected the genuine conformation of the polypeptide chain recognized by MAb DAO5. In view of the similarity of the individual complex structures (two complexes per asymmetric unit [AU] in the scFv complexes and one complex per AU in the Fab complex), we selected for further analysis those with the lowest mean temperature factor (B-factor) comprising residues 532 to 540 (Fig. 1G) (indicating the highest degree of order). Since the interactions of the two peptides with the paratope are almost identical, we will discuss the common molecular binding determinants and highlight differences only where necessary.

### Molecular determinants of DAO5 binding.

Unexpectedly, the peptide forms one α-helical turn comprising residues 535 to 539 (DVM/FLL), in stark contrast to the extended conformation observed in the cE2 structure (10). The peptide interaction with the paratope buries an area of ~730 Å^2^ on the peptide and ~650 Å^2^ on the antibody, with a total buried surface area of 1,379 Å^2^ and a shape complementarity index of 0.801 and 0.774 for the J4 and JFH-1 peptides, respectively, similar to other antibody-antigen complexes (28, 29). The B-factor and RMSD analyses indicated a stable and strong interaction with the paratope, with a higher degree of disorder toward the peptide termini (Fig. 1F and G). Of note, this segment contains two asparagine residues N532 and N540 that are glycosylated in the context of the viral particle (30). Our peptide structures show that the side chains of both asparagines are exposed in the complex and therefore able to accommodate these two N-linked glycans (Fig. S2). Interactions between the DAO5 MAb and the glycosylated protein are thus likely to be unaffected by the presence of the glycans, similar to the interactions with the peptide.

10.1128/mBio.00382-17.3 FIG S2  Compatibility of the peptide conformation with N-linked glycosylation. (A and B) Compatibility of the Fab DAO5-J4 peptide complex structure, with the positions of N-linked glycans attached to N^532^ and N^540^ of the native glycoprotein. The cartoon representation of the peptide (light orange, with the two asparagine side chains shown as sticks), colored according to atom type (blue for ND2 atoms to which the sugar chains are linked, and red for OD1 atoms). The Fab molecular surface is shown, with the heavy and light chains colored dark and light gray, respectively. Two views rotated by 180° around the indicated axis are shown. *In silico*-modeled glycan chains containing two *N*-acetylglucosamines and one mannose moiety (light blue) are displayed to demonstrate that the helical peptide conformation is compatible with two N-linked glycans being attached to the native glycoprotein. Download FIG S2, TIF file, 1.1 MB. Copyright © 2017 Vasiliauskaite et al. 2017 Vasiliauskaite et al. This content is distributed under the terms of the Creative Commons Attribution 4.0 International license.

Residues N^532^-T^534^ interact exclusively with the light chain, forming an N-terminal contact region. The α-helical turn of the peptide with residues M/F^537^, L^538^, and L^539^ establishes mainly hydrophobic side chain interactions, predominantly with the heavy chain, forming a second contact region at the C terminus of the peptide. Both side chain oxygen atoms of D^535^ form hydrogen bonds with the hydroxyl group of Y^L32^, highlighting D^535^ as an essential contact residue for DAO5 binding, whereas in the E2 structure it forms hydrogen bonds to the side chains of T^534^ and T^526^ and also to the main chain NH group of N^532^ and G^530^, thereby dominating the main chain conformation in this particular region. V^536^ does not interact with the paratope, suggesting that the interaction of D^535^ with Y^L32^ is required to form a junction connecting the two separate (N- and C-terminal) contact regions, thereby stabilizing their relative positions.

**FIG 2:**
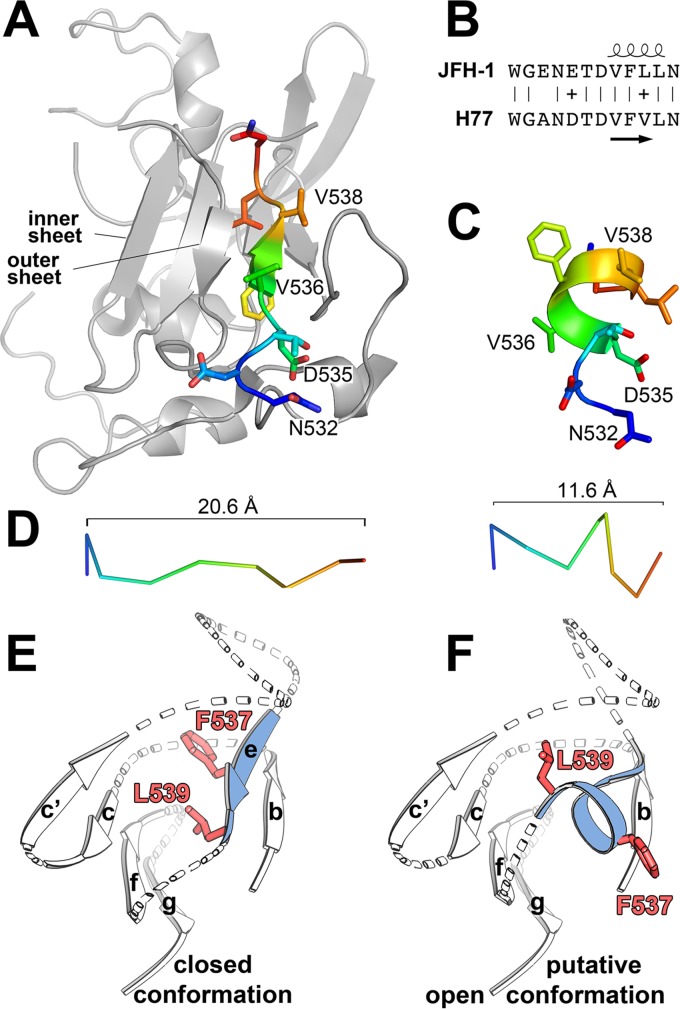
Conformation of the HCV E2 peptide aa 532 to 540. (A and C) Residues 532 to 540 of the extended conformation observed in the context of cE2 (PDB 4MWF) (A) and the helical conformation observed in the DAO5/peptide complex (C) are shown as cartoons, with the side chains shown as sticks and colored according to atom type (orange and red for oxygen and nitrogen, respectively). Carbon atoms are ramp colored from the N terminus (blue) to the C terminus (red) through green and yellow. cE2 is shown in gray. (B) Secondary structure elements taken from the crystal structures are shown above and below an amino acid alignment of the polypeptide segments from strains H77 and JFH-1. (D) Cα trace of the two backbone conformations colored as described for panel A. The extended conformation in the context of cE2 spans 20.6 Å (left panel), and the helical conformation spans 11.6 Å (right panel). (E and F) Schematic representation of the Ig-like domain within HCV E2 as observed in the E2 core fragment in the presence of a stabilizing Fab fragment (E) (PDB 4MWF) and in a putative open conformation (F) in which residues F^537^ and L^539^ (side chains are shown as red sticks) are solvent exposed, allowing for interaction with MAb DAO5, similar to the conformation of F^537^ observed in the second E2c structure (PDB 4NX3).

A detailed comparison of peptide residues 532 to 540 with the corresponding segment in the cE2 structure revealed that the short α-helix observed in complex with DAO5 folds as strand E of the β-sandwich in cE2 (Fig. 2A to C). The distance spanned by residues 532 to 540 is considerably shorter in the peptide structure than in cE2 (20.6 Å and 11.6 Å, respectively) (Fig. 2D). In the context of E2, this difference between the two conformations can likely be accommodated, because this segment is encompassed at either side by the putatively flexible C′-E and E-F loops (Fig. 2E). In the presence of nAb AR3C, the side chains of F^537^ and L^539^ are oriented toward the hydrophobic core of the β-sandwich (red side chains in Fig. 2E) (10), whereas both residues are buried to more than 80% in the DAO5 binding interface (Fig. 1E), indicating that they need to be solvent exposed to allow interaction with DAO5 (Fig. 2F). Of note, in the absence of a stabilizing neutralizing Fab fragment bound at this surface, the side chain of F^537^ is also solvent exposed (9). These results indicate that strand E is not only able to switch secondary structure upon binding of a specific antibody, but also that side chains can flip inside-out, suggesting that they are not stably anchored in the hydrophobic core of the Ig-like domain. In view of DAO5 binding to the soluble E2 ectodomain as well as infectious HCVpp and HCVcc particles (see below), we can therefore conclude that the Ig-like domain within HCV E2 can accommodate conformational rearrangements to allow for the conversion between a closed conformation and a putative open conformation. In the closed conformation, residues 536 to 538 form β-strand E (Fig. 2E), whereas the putative open conformation allows these residues to be recognized in the form of a short α-helix (Fig. 2F), with the side chains of F^537^ and L^539^ being accessible for interaction with the antibody. The high degree of sequence conservation within this region (Fig. 2B) suggests that this conformational flexibility reflects an intrinsic property of HCV E2, in line with the previously reported experimental evidence for the structural flexibility of E2 in the context of a soluble ectodomain and infectious viral particles (11, 12, 21–24).

### The Ig-like domain within soluble E2 adopts more than one conformation.

**FIG 3:**
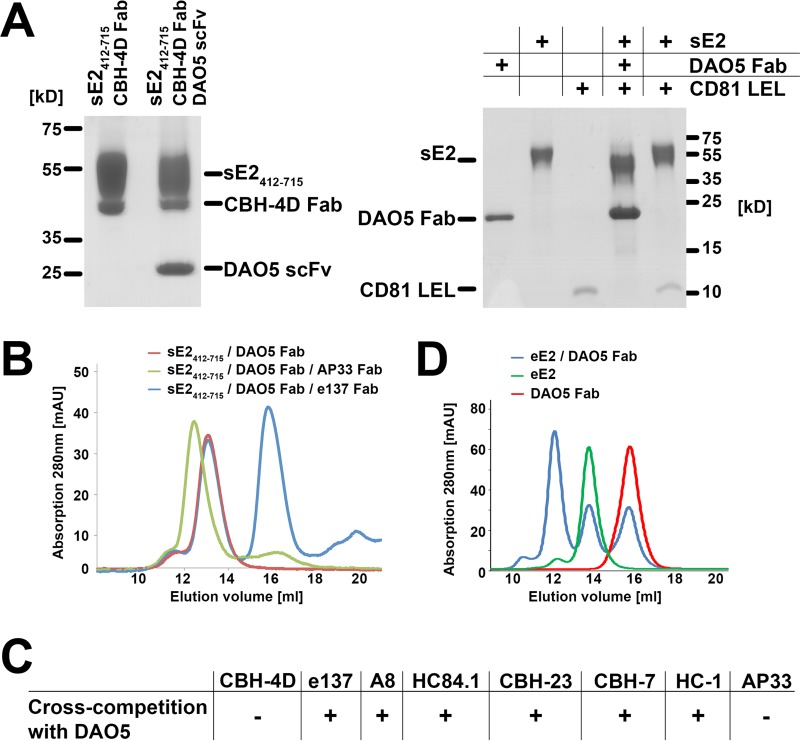
Cross-competition profile of DAO5. (A) Cross-competition and biochemical analysis of the sE2_412-715_–DAO5 complex. (Left) sE2_412-715_ and an sE2_412-715_–DAO5 scFv complex were affinity loaded onto a StrepTactin column, a CBH-4D Fab fragment was added, and the eluted complex was analyzed by SDS-PAGE under nonreducing conditions. (Right) CD81-LEL was incubated overnight at RT with the full-length HCV ectodomain (comprising residues 384 to 715; sE2) and the sE2-DAO5 Fab complex, followed by SEC and analysis of the peak fractions by SDS-PAGE under reducing conditions. DAO5 heavy and light chains form an apparent single band in the reducing gel due to an almost identical molecular mass of ~24 kDa. (B) A preformed sE2_412-715_–DAO5 Fab complex was incubated in the absence or presence of a Fab fragment targeting a non-overlapping (AP33) or overlapping (e137) region of cE2 and analyzed by SEC. After preincubation with AP33 (green), appearance of a peak at a higher molecular mass indicated ternary complex formation; after preincubation with e137 Fab (blue), the presence of peaks corresponding to the binary complex (at ~13 ml) and an isolated Fab fragment (at ~16 ml) showed that no ternary complex was formed. (C) Cross-competition profile of the sE2_412-715_–DAO5 Fab complex, obtained by SEC analysis as described for panel B, with a panel of Fab fragments derived from the indicated well-characterized anti-E2 MAbs. (D) sE2_384-713_ produced in human cells (eE2) was incubated with DAO5 Fab, and then the complex as well as the individual components were analyzed by SEC. The presence of a peak at a higher molecular mass indicated binary complex formation (12 ml, blue). A considerable protein fraction eluted in peaks corresponding to uncomplexed eE2 (green) and DAO5 Fab (red).

To exclude that DAO5 recognizes an artificial denatured form of soluble E2 (sE2), we analyzed the overall conformation of a DAO5 scFv-sE2_412-715_ complex by pulldown assay using a Fab fragment derived from a conformation-sensitive human MAb, CBH-4D, which recognizes a non-overlapping epitope outside the CD81 binding site (31). A ternary complex consisting of DAO5 scFv, sE2_412-715_, and CBH-4D Fab was observed, suggesting that the overall fold of sE2_412-715_ was not affected (Fig. 3A, left panel) despite the rearrangement within the Ig-like domain.

To determine the local conformation of the CD81 binding loop, we first tested if the DAO5 Fab-sE2 complex could interact with the soluble large extracellular loop (LEL) of CD81. Purified DAO5 Fab-sE2 complex or sE2 alone was incubated with a molar excess of CD81-LEL, and then the mixture was subjected to size exclusion chromatography (SEC). SDS-PAGE analysis of the peak fractions showed that the purified DAO5 Fab-sE2 complex, unlike sE2 alone, failed to bind CD81 (Fig. 3A, right panel). A more detailed analysis was performed by mixing a prepurified complex of sE2_412-715_–DAO5 Fab individually with Fab fragments derived from several well-characterized human and murine broadly neutralizing nAbs (bnAbs) to assess cross-competition. Ternary complex formation was analyzed by SEC. We observed a shift of the peak from a binary DAO5 Fab-sE2_412-715_ complex to that from a ternary complex incorporating Fab AP33 (which recognizes E2 aa 412 to 423 [32]), indicating, as expected, no cross-competition between AP33 and DAO5 (Fig. 3B). In contrast, mixing a preformed binary DAO5 Fab-sE2_412-715_ complex with the Fab fragment of the human bnAb e137, which targets the CD81 binding loop (33), resulted in the appearance of a lower-molecular-weight peak that corresponded to an isolated Fab fragment (Fig. 3B, blue). This indicated that no ternary complex formation is possible due to cross-competition. Similarly, we observed no ternary complex with Fab fragments from A8, HC84.1, CBH-23, CBH-7, or HC-1 (Fig. 3C), suggesting that DAO5 directly cross-competes with most bnAbs targeting the CD81 binding site.

Despite directly competing with both CD81-LEL and bnAbs targeting the CD81 binding site, DAO5 Fab surprisingly lacks neutralizing activity (Fig. S1C). Numerous MAbs targeting the CD81 binding loop have been characterized, and many of them cluster in one of two groups, being either (i) MAbs from human patients that recognize conformation-sensitive epitopes and potently neutralize HCV infection (e.g., MAbs A8 [34], e137 [33], AR3C [35], CBH-5 [36], and HC-1 [37]) (reviewed in reference 3) or (ii) rodent MAbs that recognize mostly linear epitopes in the N-terminal half of the CD81 binding loop and neutralize HCV infection to a lesser extent or not at all (e.g., 3E5 [38], 9/75 and 2/64a [39], H77.31, J6.27 [40], 19D8, 21C3, 23G2, and 21D9 [41], or 1H8 [42]). These results suggest a positive correlation between a conformation-sensitive character and a neutralizing activity of antibodies directed against the CD81 binding loop and indicate that antibodies recognizing linear epitopes within this region neutralize HCV infection less potently.

**FIG 4:**
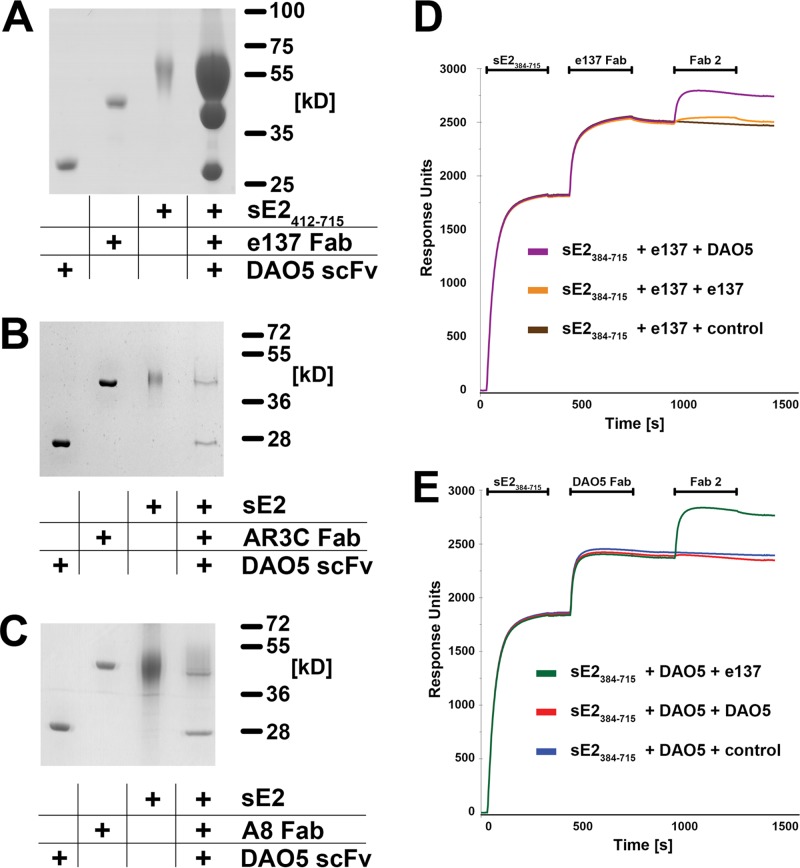
Evidence for more than one conformation of the CD81 binding loop. (A to C) sE2_412-715_ or sE2 was immobilized on a StrepTactin column and incubated first with a molar excess of DAO5 scFv and subsequently with e137 (A), AR3C (B), or A8 Fab (C). Eluted complexes were analyzed by SDS-PAGE. (D and E) Real-time SPR analysis of Fab binding to immobilized sE2 recorded the binding response (in resonance units [RU]) as a function of time. Fab fragments of e137 (D) and DAO5 (E) were injected over a surface with immobilized HCV sE2. After reaching equilibrium between association and dissociation, a second Fab fragment (either the same Fab again or the alternative one) or a buffer control was injected.

We analyzed in more detail the competition of DAO5 with bnAbs targeting the CD81 binding site, using bnAb e137 as an example. Although no ternary complex comprising sE2, e137 Fab, and DAO5 Fab was observed, fragments of both antibodies bound to sE2_412-715_ molecules immobilized on a StrepTactin column (Fig. 4A), in apparent contradiction with the SEC results presented in Fig. 3B. It is important to note, however, that SEC reveals whether a single glycoprotein molecule is able to bind both antibody fragments simultaneously, whereas the StrepTactin column pulldown assay investigates whether a glycoprotein solution contains both molecules recognized by DAO5 and other molecules recognized by e137. Similarly, immobilized sE2 molecules pulled down fragments of both DAO5 and AR3C (Fig. 4B), as well as DAO5 and A8 (Fig. 4C). These results suggest that the Ig-like domain in soluble E2 can be present in more than one conformation and therefore be recognized either by DAO5 or by one of the human bnAbs.

Although several non-overlapping conformation-dependent antibodies recognize sE2 produced in insect cells (43), a crystal structure of this protein has not yet been reported. In order to exclude a specific conformation of the insect cell-derived protein, we produced sE2_384-713_ in human cells (eE2) as described before (9) and mixed it with DAO5 Fab overnight at 4°C. SEC analysis revealed a peak corresponding to a DAO5 Fab-eE2 complex (Fig. 3D), demonstrating that the majority of eE2 adopts the putative open conformation in the Ig-like domain. The fact that a considerable fraction of eE2 did not form a complex with DAO5 Fab (Fig. 3D) supports the notion that the Ig-like domain in eE2 can also adopt more than one conformation recognized by DAO5 and putatively by AR3C/e137, respectively, similar to the insect-cell derived sE2.

To further analyze this conformational flexibility, we performed surface plasmon resonance (SPR) experiments, in which we injected two Fabs consecutively over immobilized sE2 (Fig. 4D and E). Nearly equal signals resulted from binding of e137 Fab (Fig. 4D) and DAO5 Fab (Fig. 4E), indicating that comparable numbers of immobilized sE2 molecules were recognized by either Fab. After reaching an equilibrium between association and dissociation of the first Fab, a repeated injection of the same Fab did not lead to a further increase in binding, indicating that saturation was reached after the first injection. In contrast, injection of the second Fab resulted in markedly increased binding to sE2, supporting the hypothesis that sE2 in more than one conformation was immobilized. After binding of the second Fab, the total amount of bound Fab reached similar levels, regardless of which Fab was bound first. The fact that DAO5 recognizes E1E2 complex expressed in HEK cells (Fig. S1A and B) and a soluble E2 ectodomain expressed in either insect or mammalian cells with a similar binding efficiency suggests that the conformational flexibility in the Ig-like domain is independent of the presence of HVR1 and the expression/host type.

### More than one conformation of E2 is present simultaneously on the surface of infectious HCV particles.

**FIG 5:**
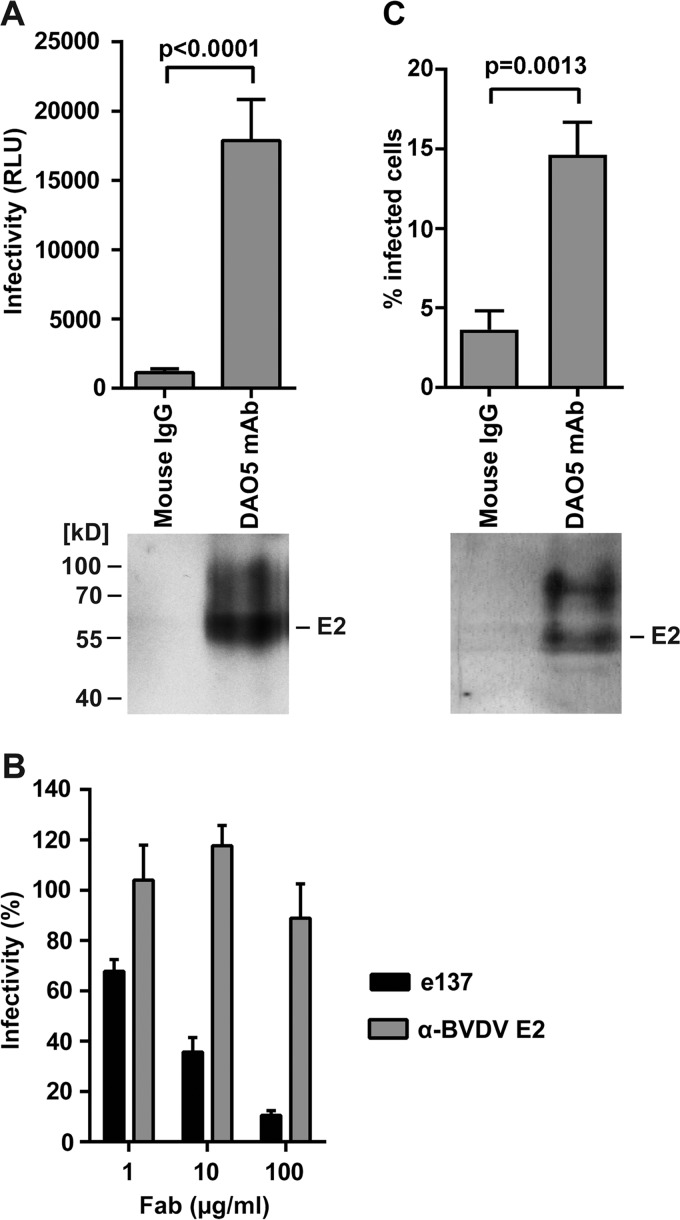
More than one conformation of the Ig-like domain was observed on both HCVpp and HCVcc infectious particles. (A) Concentrated HCVpp were immunocaptured using Immuno tubes. After removing unbound material, the captured particles were analyzed by SDS-PAGE, followed by immunoblotting to detect E2 (lower panel). The presence of captured infectious HCVpp was determined by overnight incubation with Huh7 cells in suspension. The cells were then transferred into tissue culture dishes, and infectivity levels were determined 48 h later by measuring luciferase activity, shown as relative light units (RLU) (upper panel). (B) DAO5-captured particles were incubated with Huh7 cells as described for panel A in the presence of e137 Fab or control anti-bovine viral diarrhea virus (BVDV) E2 Fab at the indicated concentrations. Infectivity levels were determined as described above and are shown as the percent infectivity in the absence of Fab. (C) Concentrated HCVcc were immunocaptured as described for panel A. After removing unbound material, captured particles were analyzed by SDS-PAGE followed by immunoblotting to detect E2 (lower panel). The presence of captured infectious HCVcc was determined by overnight incubation with Huh7 cells in suspension. The cells were then transferred into tissue culture dishes, and the number of infected cells was determined after 72 h by flow cytometry after labeling with anti-NS5A MAb 9E10 (upper panel). Experiments were performed at least in triplicate, and results shown are means ± standard deviations. The *P* values (panel A and C) were calculated using an unpaired *t* test.

The most sensitive way to detect interactions between nAbs and virus particles is to measure neutralization rather than direct protein-protein interactions. This was not possible with MAb DAO5, as it is a non-neutralizing antibody. Nevertheless, given that all experiments described so far were carried out using a soluble ectodomain of E2, it was crucial to demonstrate that more than one conformation is found in the functional full-length E1E2 glycoprotein complex at the virus surface. We therefore tested whether DAO5 MAb binds to the retrovirus-based HCV pseudoparticles (HCVpp) displaying genotype 2a JFH1 E1E2. DAO5 MAb was immobilized on Immuno tubes and incubated with the luciferase reporter HCVpp, and then the captured particles were tested for infectivity and E2 content. We found that DAO5 specifically captured full-length E2 incorporated into infectious HCVpp, demonstrating that its epitope was displayed at the surface of these particles (Fig. 5A). To investigate whether more than one conformation of E2 is present simultaneously at the virus surface, we tested the neutralization of immobilized particles by using the cross-competing e137 Fab. Our results showed that the particles captured by the DAO5 MAb were efficiently neutralized by e137 Fab in a concentration-dependent manner (Fig. 5B), suggesting that more than one conformation of the Ig-like domain is simultaneously present on the surface of the same HCVpp.

A key experiment investigated whether the DAO5 epitope can also be detected at the surface of infectious cell culture-derived particles (HCVcc). It has been reported that antibodies targeting the viral glycoproteins do not efficiently bind HCVcc (44). However, modified immunocapture experiments using HCVcc revealed that DAO5 specifically captured full-length E2 (Fig. 5C, lower panel). In addition, immunocapture of HCVcc using DAO5 resulted in ~15% infected cells, whereas immunocapture using a control antibody only yielded ~4% infected cells (Fig. 5C, upper panel), demonstrating that the DAO5 epitope is displayed at the surface of infectious HCVcc. These data support the notion that the Ig-like domain within functional full-length E1E2 glycoprotein complexes can adopt either conformation at the surface of infectious virus particles.

## DISCUSSION

In this study, we found that an important part of the CD81 binding site within the HCV glycoprotein E2, the CD81 binding loop, adopts more than one distinct conformation at the surface of both HCVpp and HCVcc. One of these conformations has been observed in complex with the human bnAb AR3C (10); insights into another one are provided by our crystal structure of MAb DAO5 in complex with an epitope peptide. This murine MAb was derived from immunization with a soluble E2 ectodomain produced in insect cells that binds several conformation-sensitive antibodies (43) and elicits broadly neutralizing, conformation-sensitive antibodies (45). Biochemical analyses demonstrated that three human bnAbs bind to a conformation that is different from the open conformation recognized by DAO5. It thus appears that the majority of bnAbs target a closed conformation of the Ig-like domain with particular relevance for the “receptor binding” step during virus entry, although further structural studies are required to investigate this hypothesis.

Of note, HCV envelope glycoprotein complexes at the virus surface are not readily accessible to antibodies, either due to their low abundance or occlusion by host-derived lipoproteins (44). Our results indicate that functional full-length E1E2 glycoprotein complexes can be trapped in two different conformations at the surface of HCVpp and HCVcc particles by MAbs DAO5 and e137, with residues that are part of the hydrophobic core in one conformation being solvent exposed in the second conformation. A similar structural promiscuity has been described for the AB loop of tear lipocalin, a lipid binding protein in tears (46), and in this context it is tempting to speculate whether the exposure of hydrophobic side chains in E2 is related to a putative lipid binding function and the extensive association of HCV virions with host lipoproteins.

The observed structural differences within the Ig-like domain of HCV E2 are striking (Fig. 2E and F) and further illustrate how structurally flexible this molecule is. Our results extend the previously described notion that segments involved in the CD81 binding site are conformationally flexible (21, 24, 25). Remarkably, not only was the interaction site within E2 shown to be structurally flexible, but also marked conformational fluctuations were demonstrated for the region within the CD81-LEL, which is thought to interact with E2 (47). This observation, together with the above-mentioned structural flexibilities in the CD81 binding site, suggests that the currently available structures of E2 might not provide an accurate view of its conformation when bound to CD81. It is likely that binding of a given CD81 binding loop conformation to nAbs correlates with its binding to CD81, and the fact that the putative open conformation is not recognized by nAbs suggests that it does not bind to CD81. However, a high-resolution structure of E2 in complex with CD81 is required to understand the molecular determinants of this interaction.

The fact that DAO5 does not neutralize HCV infection despite the demonstrated cross-competition with CD81 binding implies that within the virus envelope the protein cannot be converted from one conformation to the other. This suggests that during virion assembly both conformations are incorporated into the virus envelope, and it raises the question why a receptor binding glycoprotein like HCV E2 is so conformationally flexible, i.e., how can the virus benefit from displaying a receptor binding site in different conformations? One possibility is that the helical conformation of the Ig-like domain serves as a decoy that distracts the host immune system from the relevant, receptor binding conformation. In this context, it will be interesting to investigate whether antibodies recognizing the helical conformation of the CD81 binding loop are present in patient serum, e.g., by using an epitope scaffold carrying a conformation-restricted DAO5 epitope.

The described flexibility also suggests that a structure-guided stabilization of the Ig-like domain within E2, e.g., by engineering additional disulfide bonds, can potentially improve the capacity of E2 to induce neutralizing antibodies by eliminating conversion to a particular conformer. Our results therefore provide novel insights regarding the interplay between E2 and the host humoral immune response, and understanding this interplay will greatly aid the rational design of an effective HCV vaccine.

## MATERIALS AND METHODS

### Antibodies.

The mouse MAb DAO5 was generated and its epitope mapped as described in Text S1. Purified DAO5 IgG was labeled with biotinamidohexanoic acid 3-sulfo-*N*-hydroxysuccinide ester (BAC-SulfoNHS) using the BiotinTag Micro biotinylation kit (Sigma).

### Retrovirus-based HCV pseudoparticles.

Human epithelial kidney cells (HEK-293T; ATCC CRL-1573) and human hepatoma Huh7 cells were propagated as reported previously (48). HCVpp were generated in HEK-293T cells (49). Virus particles were filtered through 0.45-µm-pore-size membrane and concentrated 10-fold using Amicon Ultra-15 centrifugal units with a 100,000 molecular weight cutoff membrane. Huh7 cells were infected with MAb DAO5-captured HCVpp as described below.

### Immunocapture of infectious HCV particles (HCVpp/HCVcc).

Nunc Immuno tubes were coated with 40 µg/ml MAb DAO5 IgG or mouse IgG control (M-9035; Sigma) in phosphate-buffered saline (PBS) overnight, blocked with PBS containing 5 to 10% fetal calf serum for 3 h at room temperature, and then rinsed thoroughly with PBS. Concentrated HCVpp or concentrated HCVcc of infectious HCV strain JFH-1 was added to the tubes and incubated for at least 3 h at room temperature (RT). Unbound material was removed by rinsing thoroughly with PBS, and the immunocaptured material was analyzed as follows.

First, to test for infectivity, 1 × 10^5^ Huh7 cells were added to each tube and incubated overnight at 37°C. The human hepatoma cell line Huh7 was a kind gift from Jean Dubuisson and has been described previously (48). The cells were then seeded into a 12-well cell culture plate and incubated for a further 48 h (for HCVpp) or 72 h (HCVcc). To determine HCVpp infectivity, cells were lysed and luciferase activity was measured using Bright Glo (Promega). To test for neutralization, the immunocaptured HCVpps were preincubated with e137 and control Fabs in cell culture medium for 1 h before adding cells. To determine HCVcc infectivity, cells were detached using cell dissociation buffer (Sigma), fixed with 2% formaldehyde for 20 min, washed with PBS, and then permeabilized with PBS containing 0.1% saponin (Sigma) for 30 min on ice. After washing with PBS containing 0.02% Tween 20 (PBS-T; Sigma), the cells were incubated on ice for 1 h with the anti-HCV NS5A MAb 9E10 (a kind gift of Charles M. Rice) or an irrelevant isotype control IgG, washed with PBS-T, and then incubated further with Alexa Fluor 488 donkey anti-mouse IgG H+L. Following washing with PBS-T, the stained cells were resuspended in PBS and analyzed by flow cytometry on a Guava EasyCyte HT cell sorter (Merck Millipore), and virus infectivity was measured as the percentage of infected cells.

Next, to test for HCV E2 content, proteins were stripped from the Nunc Immuno tubes by adding Laemmli sample buffer containing β-mercaptoethanol and heating at 100°C for 3 min. Proteins were separated by 10% SDS-PAGE, followed by immunoblotting for HCV E2 with biotinylated MAb DAO5 and a streptavidin-horseradish peroxidase.

### Ethics statement.

The mouse experiments were approved by the University of Glasgow Ethical Review Process and were conducted following guidelines from the National Centre for the Replacement, Refinement and Reduction of Animals in Research (NC3R) under project license 60/3657 granted by the UK Home Office Animals (Scientific Procedures) Act 1986.

### Recombinant proteins, complex formation, crystallization, and structure determination.

The generation and purification of recombinant DAO5 Fab or scFv and the HCV E2 soluble ectodomains (sE2) produced in insect cells as well as crystallization, data collection, processing, structure determination, and analysis are described in detail in Text S1 in the supplemental material. The mammalian HCV E2 ectodomain (residues 384 to 713) was produced in HEK 293 cells as described before (9). Space groups and cell dimensions of the crystals, resolution limits, data collection details, and refinement statistics are summarized in Table S1.

10.1128/mBio.00382-17.4 TABLE S1  Diffraction data and refinement statistics. Download TABLE S1, DOCX file, 0.1 MB. Copyright © 2017 Vasiliauskaite et al. 2017 Vasiliauskaite et al. This content is distributed under the terms of the Creative Commons Attribution 4.0 International license.

### Cross-competition analysis of antibody fragments and CD81 by SEC

sE2 or sE2_412-715_ were mixed together with DAO5 Fab for 16 h at 30°C to allow for complex formation, and the complex was subsequently purified by SEC. For Fab cross-competition analysis, a second Fab was added to the purified complex for 16 h at 4°C, followed by a second SEC analysis on a Superdex 200 10/300 column. Soluble CD81-LEL was produced as described elsewhere (50). One hundred micrograms of sE2 or purified sE2-DAO5 Fab complex was mixed with a molar excess of CD81-LEL overnight at 23°C, followed by SEC analysis on a Superdex 200 10/300 column. Peak fractions were concentrated and analyzed by SDS-PAGE under nonreducing conditions. followed by Coomassie blue staining.

### Pulldown assay of antibody fragments using a soluble E2 ectodomain.

For pulldown assays, sE2_412-715_ containing a Strep tag was used. The sE2_412-715_–DAO5 Fab or sE2_412-715_–DAO5 scFv complexes were purified as described above, bound to a StrepTactin superflow minicolumn, and washed with 10 column volumes followed by addition of a molar excess of CBH-4D Fab and another washing step. To analyze cross-competition between e137, AR3C, and DAO5 antibody fragments, sE2 or sE2_412-715_ was bound to a StrepTactin Superflow minicolumn and washed with 10 column volumes, followed by addition of the first antibody fragment (from which the Strep tag had been proteolytically removed), washing, addition of the second antibody fragment (also lacking the Strep tag), and another washing step. Complexes were eluted, and all fractions were analyzed by SDS-PAGE followed by Coomassie blue staining.

### Surface plasmon resonance analysis.

SPR experiments were performed in triplicate on a Biacore T200 system (GE Healthcare, Uppsala, Sweden), equilibrated at 25°C in PBS (pH 7.4) complemented with 0.01% Tween 20, using a CM5 sensor chip with a density of around 13,000 response units (RU; similar to measurement in picrograms per square millimeter) and anti-Strep antibody (C23.21) immobilized through amide bonds.

sE2_412-715_ (5 µg/ml) was captured on the chip via its Strep tag at a density of 2,100 to 2,200 RU. The DAO5 or e137 Fabs (50 µg/ml) were then injected for 5 min, followed by buffer, DAO5, or e137. The Fab signal monitored on an anti-Strep reference surface was subtracted.

### Accession number(s).

The atomic coordinates and structure factors for three structures have been deposited in the Protein Data Bank (http://www.pdb.org/) under accession numbers 5NPH (Fab/J4), 5NPI (scFv/J4), and 5NPJ (scFv/JFH).
