# Supplementary material for: Conformational Flexibility in the Immunoglobulin-Like Domain of the Hepatitis C Virus Glycoprotein E2
Source: mBio. 2017 May 16;8(3):e00382-17. doi: 10.1128/mBio.00382-17 (PMC5433095; doi:10.1128/mBio.00382-17)
Supplement: TEXT S1 [file mbo003173308s1.docx]

## Table of contents

|  | **Page** |
| --- | --- |
| Supplementary Data: | 2 |
| Supplementary Methods: | 4 |
| Supplementary References: | 9 |

## Supplementary data:

### Generation and characterization of the DAO5 mAb

To generate mAbs targeting conserved regions of the HCV E2 glycoprotein, we immunized mice with purified sE2 of different genotypes (gts) as detailed below in Supplementary Methods. Standard hybridoma technology was then used to produce a panel of novel anti-E2 mAbs, among them DAO5.

Initial characterization showed that DAO5 bound E2 from gts 2, 3 and 4 (but not 1a, 5 or 6) recognizing a linear epitope that was mapped by phage peptide display library screen to the sequence ETDVFLLN, corresponding to residues 533 to 540 in gt 2a JFH1 (data not shown). The mapping was consolidated by testing a series of peptides for their ability to compete with E2 (gt 2a JFH1) for binding to DAO5 in competition ELISA. Three overlapping peptides corresponding to the gt 1b J4 sequence of the epitope were able to compete with E2, although a peptide with E^533^ at the very N-terminus was less effective than the other two, indicating that the DAO5 epitope includes one or more residues upstream of E^533^ (S1A Fig.). Peptides corresponding to the 1a H77 sequence had no effect, suggesting that either E^533^ or L^538^ - the two residues within the epitope that differ between J4/JFH1 and H77 - are critical for binding (S1A Fig.).

The epitope corresponds to the C-terminal part of the CD81 binding loop and contains D^535^, a highly conserved residue that acts as a contact residue for CD81 and various bnAbs [1, 2]. We confirmed binding of DAO5 to D^535^ by GNA capture ELISA using previously described mutants of E2 in which this residue or the adjacent W^529^ (upstream of the DAO5 epitope) was substituted with alanine in the context of full length E1E2 [3] (S1B Fig.). As controls, we used two well-characterized mAbs, AP33 and HC-1. AP33, which recognizes aa412-423 [4], was not affected by mutation of D^535^, whereas HC-1, which requires both W^529^ and D^535^ for binding [5], did not recognize either of the mutants (S1B Fig.). Given that DAO5 binds to D^535^, an essential residue for binding of CD81 and numerous bnAbs, we tested the ability of DAO5 to neutralize virus infection. Surprisingly, neither DAO5 mAb nor DAO5 Fab affected infectivity of HCVpp or HCVcc (S1C Fig.), although they bind to D^535^, a residue that is critical for CD81 interaction.

## Supplementary Methods:

### Antibodies.

The anti-E2 mouse mAb AP33 and human mAb HC-1 have been described before [6, 7]. An irrelevant mouse mAb A164 was used as isotype control.

### Generation of anti-E2 mouse monoclonal antibodies.

Balb/c mice were immunized essentially as described [6] with purified soluble HCV E2_384-715_ ectodomains of different genotypes (gt). A primary subcutaneous immunization with 15 µg of gt 2a JFH1 emulsified in Freund’s Complete Adjuvant was followed by three 15 µg boosters with gts 2b, 4 and 5 emulsified in Freund’s Incomplete Adjuvant. After intra-peritoneal challenge with 75 µg of gt 1b, spleen cells were fused with myeloma cells and hybridomas selected using HAT medium. Hybridoma supernatants were screened for E2 reactivity by GNA ELISA and positive clones were purified by limiting dilution to produce nine stable monoclonal hybridoma cell lines, designated DAO1 to DAO9. Monoclonal antibodies (mAb) were affinity purified on protein G.

### Generation of cell culture infectious HCV (HCVcc).

Infectious HCVcc strain JFH-1 was generated following elctroporation of in vitro-transcribed viral genomic RNA into Huh7 cells as described (Wakita et al., 2005). Virus particles were filtered through 0.45 µm pore-size membrane. To test for antibody-mediate neutralization, Huh7-J20 reporter cells seeded into 96-well tissue culture plates were infected with HCVcc that had been pre-incubated for 1 hr with appropriate antibody (whole IgG or Fab) and the virus infectivity levels determined by measuring the secreted alkaline phosphatase (SEAP) activity in the culture medium [8].

### GNA-capture and peptide competition ELISAs.

The enzyme-linked immunosorbent assay (ELISA) to detect mAb binding to E2 glycoprotein was performed essentially as described previously (Patel et al, 2000). WT and mutant genotype 2a JFH1 E1E2 proteins were made by transfecting HEK-293T cells with the appropriate expression plasmids. Two days after transfection, cells were washed in phosphate-buffered saline (PBS) and resuspended in lysis buffer (40 mM Tris pH 7.5, 1 mM EDTA, 150 mM NaCl, 1% Igepal CA-630, 20 mM iodoacetamide, and complete protease inhibitor cocktail). Nuclei were pelleted by centrifugation at 15,000 xg, and the cytoplasmic extract stored in aliquots at -20°C. Microtiter plates (Immulon II) coated with 2.5 µg/ml of Galanthus nivalis agglutinin (GNA) were used to capture E2 glycoproteins from cell lysates. PBS containing 0.02% Tween 20 (PBST) was used for all washing steps and dilution of reagents. Bound glycoprotein was detected using either (a) native anti-E2 mAbs followed by anti-mouse horseradish peroxidase conjugate (Sigma-Aldrich), or (b) biotinylated anti-E2 mAbs followed by streptavidin horseradish peroxidase polymer (Sigma-Aldrich) and then 3,3’,5,5’-tetramethylbenzidine (TMB) substrate. Color development was stopped with 0.5M H_2_SO_4_ and absorbance values measured at 450 nm.

Peptides for competition analysis were selected from overlapping arrays spanning HCV E2 obtained through the NIH Biodefense and Emerging Infections Research Resources Repository, NIAID, NIH (1a H77: NR-3749; 1b J4: NR-3739). DAO5 mAb (0.05 µg/ml) was mixed with an equal volume of serially diluted peptide, incubated at RT for 30 mins and then transferred to E2 captured on GNA. After incubation for 1 hr at room temperature, bound antibody was detected with anti-mouse HRP conjugate.

### Production and purification of recombinant proteins.

The coding regions of MAb DAO5 were amplified by RT-PCR using total RNA derived from the DAO5 hybridoma cell line as template and degenerate heavy and light chain primer pairs (primer sequences available on request). Following determination of the nucleotide sequence of the PCR products, codon-optimized synthetic genes for heavy and light chains were synthesized (Genscript, Piscataway, USA) and cloned into *Drosophila melanogaster* S2 expression vectors for Fab or scFv described previously [9, 10]. The HCV E2 full-length soluble ectodomains (sE2) of strains JFH-1, Con1 and the clinical isolates UKN2b_2.8, UKN4_11.1 and UKN5_14.4 used for immunization and the UKN2b_2.8 ectodomain lacking the hypervariable region 1 (sE2_412-715_) were expressed in *Drosophila* S2 cells as previously described [11, 12]. Briefly, *Drosophila* S2 cells were transfected as reported previously [13], amplified, and induced with 4 μM CdCl2 at a density of ~8x 10^6^ cells/ml for 6-9 days for large-scale production. Proteins were purified from the supernatant by affinity chromatography using a Strep-Tactin Superflow column (IBA, Goettingen, Germany) followed by size exclusion chromatography (SEC) using a Superdex200 column (GE Healthcare, Uppsala, Sweden). Pure monomeric proteins were concentrated to ~20 mg/ml. For co-crystallization purposes the affinity tag of the DAO5 Fab was removed by enterokinase digestion (EKMax, Invitrogen, San Diego, USA) according to the manufacturer’s instructions followed by removal of uncleaved protein using Strep-Tactin affinity chromatography and a second SEC. The pure, cleaved protein was concentrated to ~25 mg/ml.

### Complex formation and crystallization.

Synthetic peptides comprising E2 residues 529-540 of the HCV strains J4 (WGENETDVMLLN) and JFH-1 (WGENETDVFLLN) were purchased from GenScript and dissolved in 20 mM Tris pH 9.0. DAO5 Fab was complexed with peptide at a molar ratio of 1:6 overnight at 277K (10 mg/ml Fab + 1.72 mg/ml peptide). Crystals of the Fab-peptide complex were grown at 293 K by using the hanging-drop vapor diffusion method with drops containing 1 µl complex solution (11.72 mg/ml in 20 mM Tris [pH 9.0]) mixed with 1 µl reservoir solution containing 20% PEG3350 and 200 mM sodium thiocyanate and flash-frozen in liquid nitrogen after transferring crystals to a cryo-protective solution containing the mother liquor and 20% (v/v) glycerol. Crystals of DAO5 scFv were obtained by hanging-drop vapour diffusion method in drops containing 1 µl DAO5 scFv (9 mg/ml in in 10 mM TRIS pH 8.0, 150 mM NaCl) and 1 µl of reservoir solution (29% PEG 400, 200 mM MES pH 6.5 and 150 mM sodium acetate). Peptides were soaked into these crystals by transferring the crystals to mother liquor supplemented with 0.1 mM peptide for 5 hr (J4) or overnight (JFH-1) at 298K and were directly flash-frozen in liquid nitrogen.

### X-ray data collection, structure determination and refinement.

Data were collected at the Synchrotron Soleil beamline Proxima-1 and the Swiss Light source beamline PX-I. X-ray diffraction data were processed, scaled, and reduced by using XDS [14], Pointless [15], and programs from the CCP4 suite [16]. The structure of the DAO5 scFv was determined by the molecular replacement method using Phaser [17]. We used separate variable and constant regions of a hypothetical scFv assembled from the best sequence match in the Protein Data Bank (PDB), the light chain (LC) reported under PDB accession number 1FH5 and the heavy chain (HC) reported under PDB accession number 3RHV, as a search model for the scFv and the refined scFv and a mouse IgG1 constant region as search model for the Fab fragment. Manual model building was carrried out using Coot [18] and refinement was done using AutoBuster [19]. Peptides were omitted in the initial building cycles until refinement of the antibody fragments was finished to obtain high-quality unbiased difference maps for building of the peptides.

Multiple structural superposition of the peptides from three crystal structures and calculation of the root mean square deviation (RMSD) was performed using the SuperPose server [20]. Pairwise comparison of crystal structures was performed by superposition of the corresponding peptides and RMSD calculation using Chimera [21]. Surface complementarity coefficients were calculated using programs from CCP4 suite [16]. Electrostatic potentials were calculated using the adaptive Poisson Boltzmann solver [22] and protein-protein interactions were identified using the protein interaction calculator (PIC) [23]. Composite omit maps were generated using CNSsolve [24]. The buried surface area within the complex was determined using the PISA server [25]. Figures were prepared in PyMol ([www.pymol.org](http://www.pymol.org)).

## Supplementary References:

1. Owsianka AM, Timms JM, Tarr AW, Brown RJ, Hickling TP, Szwejk A, et al. Identification of conserved residues in the E2 envelope glycoprotein of the hepatitis C virus that are critical for CD81 binding. J Virol. 2006;80(17):8695-704. PubMed PMID: 16912317.

2. Rothwangl KB, Manicassamy B, Uprichard SL, Rong L. Dissecting the role of putative CD81 binding regions of E2 in mediating HCV entry: putative CD81 binding region 1 is not involved in CD81 binding. Virology journal. 2008;5:46. PubMed PMID: 18355410.

3. Witteveldt J, Evans MJ, Bitzegeio J, Koutsoudakis G, Owsianka AM, Angus AGN, et al. CD81 is dispensable for hepatitis C virus cell-to-cell transmission in hepatoma cells. J Gen Virol. 2009;90(Pt 1):48-58. doi: 10.1099/vir.0.006700-0. PubMed PMID: 19088272.

4. Owsianka A, Clayton RF, Loomis-Price LD, McKeating JA, Patel AH. Functional analysis of hepatitis C virus E2 glycoproteins and virus-like particles reveals structural dissimilarities between different forms of E2. J Gen Virol. 2001;82(Pt 8):1877-83. PubMed PMID: 11457993.

5. Keck Z-Y, Saha A, Xia J, Wang Y, Lau P, Krey T, et al. Mapping a Region of Hepatitis C Virus E2 That Is Responsible for Escape from Neutralizing Antibodies and a Core CD81-Binding Region That Does Not Tolerate Neutralization Escape Mutations. Journal of Virology. 2011;85(20):10451-63. doi: 10.1128/JVI.05259-11. PubMed PMID: 21813602.

6. Clayton RF, Owsianka AM, Aitken J, Graham S, Bhella D, Patel AH. Analysis of antigenicity and topology of E2 glycoprotein present on recombinant hepatitis C virus-like particles. Journal of Virology. 2002;76(15):7672. PubMed PMID: 11930429548064525344related:IMw1vnZmkaUJ.

7. Keck Z-Y, Olson O, Gal-Tanamy M, Xia J, Patel AH, Dreux M, et al. A point mutation leading to hepatitis C virus escape from neutralization by a monoclonal antibody to a conserved conformational epitope. Journal of Virology. 2008;82(12):6067-72. doi: 10.1128/JVI.00252-08. PubMed PMID: 18385242; PubMed Central PMCID: PMCPMC2395135.

8. Iro M, Witteveldt J, Angus AG, Woerz I, Kaul A, Bartenschlager R, et al. A reporter cell line for rapid and sensitive evaluation of hepatitis C virus infectivity and replication. Antiviral Res. 2009;83(2):148-55. doi: 10.1016/j.antiviral.2009.04.007. PubMed PMID: 19397930.

9. Backovic M, Johansson DX, Klupp BG, Mettenleiter TC, Persson MAA, Rey FA. Efficient method for production of high yields of Fab fragments in Drosophila S2 cells. Protein Eng Des Sel. 2010;23(4):169-74. doi: 10.1093/protein/gzp088. PubMed PMID: 20100703.

10. Gilmartin AA, Lamp B, Rümenapf T, Persson MAA, Rey FA, Krey T. High-level secretion of recombinant monomeric murine and human single-chain Fv antibodies from Drosophila S2 cells. Protein engineering, design &amp; selection : PEDS. 2011:1-8. doi: 10.1093/protein/gzr058.

11. Krey T, d'Alayer J, Kikuti CM, Saulnier A, Damier-Piolle L, Petitpas I, et al. The disulfide bonds in glycoprotein E2 of hepatitis C virus reveal the tertiary organization of the molecule. PLoS Pathog. 2010;6(2):e1000762. Epub 2010/02/23. doi: 10.1371/journal.ppat.1000762. PubMed PMID: 20174556; PubMed Central PMCID: PMC2824758.

12. Tarr AW, Lafaye P, Meredith L, Damier-Piolle L, Urbanowicz RA, Meola A, et al. An alpaca nanobody inhibits hepatitis C virus entry and cell-to-cell transmission. Hepatology. 2013;58(3):932-9. doi: 10.1002/hep.26430. PubMed PMID: 23553604.

13. Johansson DX, Krey T, Andersson O. Production of Recombinant Antibodies in Drosophila melanogaster S2 Cells. Methods in molecular biology (Clifton, NJ). 2012;907:359-70. doi: 10.1007/978-1-61779-974-7_21. PubMed PMID: 22907363.

14. Kabsch W. Automatic indexing of rotation diffraction patterns. J Appl Crystallogr. 1988;(21):67-72.

15. Evans P. Scaling and assessment of data quality. Acta Crystallogr D Biol Crystallogr. 2005;62(Pt 1):72-82. Epub 2005/12/22. doi: S0907444905036693 [pii] 10.1107/S0907444905036693. PubMed PMID: 16369096.

16. Collaborative Computational Project. The CCP4 suite: programs for protein crystallography. Acta Crystallogr D Biol Crystallogr. 1994;50(Pt 5):760-3. Epub 1994/09/01. doi: 10.1107/S0907444994003112 S0907444994003112 [pii]. PubMed PMID: 15299374.

17. McCoy AJ, Grosse-Kunstleve RW, Adams PD, Winn MD, Storoni LC, Read RJ. Phaser crystallographic software. J Appl Crystallogr. 2007;40(Pt 4):658-74. Epub 2007/08/01. doi: 10.1107/S0021889807021206. PubMed PMID: 19461840; PubMed Central PMCID: PMC2483472.

18. Emsley P, Lohkamp B, Scott WG, Cowtan K. Features and development of Coot. Acta Crystallogr D Biol Crystallogr. 2010;66(Pt 4):486-501. Epub 2010/04/13. doi: S0907444910007493 [pii] 10.1107/S0907444910007493. PubMed PMID: 20383002; PubMed Central PMCID: PMC2852313.

19. Bricogne G, Blanc E, Brandl M, Flensburg C, Keller P, Paciorek P, et al. BUSTER version 2.9. 2010:Cambridge, United Kingdom, Global Phasing Ltd.

20. Maiti R, Van Domselaar GH, Zhang H, Wishart DS. SuperPose: a simple server for sophisticated structural superposition. Nucleic acids research. 2004;32(Web Server issue):W590-4. doi: 10.1093/nar/gkh477. PubMed PMID: 15215457; PubMed Central PMCID: PMCPMC441615.

21. Pettersen EF, Goddard TD, Huang CC, Couch GS, Greenblatt DM, Meng EC, et al. UCSF Chimera--a visualization system for exploratory research and analysis. Journal of Computational Chemistry. 2004;25(13):1605-12. doi: 10.1002/jcc.20084. PubMed PMID: 15264254.

22. Baker NA, Sept D, Joseph S, Holst MJ, McCammon JA. Electrostatics of nanosystems: application to microtubules and the ribosome. Proc Natl Acad Sci USA. 2001;98(18):10037-41. doi: 10.1073/pnas.181342398. PubMed PMID: 11517324.

23. Tina KG, Bhadra R, Srinivasan N. PIC: Protein Interactions Calculator. Nucleic acids research. 2007;35(Web Server issue):W473-6. doi: 10.1093/nar/gkm423. PubMed PMID: 17584791; PubMed Central PMCID: PMCPMC1933215.

24. Brunger AT, Adams PD, Clore GM, DeLano WL, Gros P, Grosse-Kunstleve RW, et al. Crystallography &amp; NMR System: A New Software Suite for Macromolecular Structure Determination. Acta Crystallographica Section D: Biological Crystallography. 1998;54(5):905-21. doi: 10.1107/S0907444998003254. PubMed PMID: 9218492265354748952related:GOQnqd-p7n8J.

25. Krissinel E, Henrick K. Inference of macromolecular assemblies from crystalline state. Journal of molecular biology. 2007;372(3):774-97. Epub 2007/08/08. doi: 10.1016/j.jmb.2007.05.022. PubMed PMID: 17681537.
