# Supplementary figures and images for: Conformational Flexibility in the Immunoglobulin-Like Domain of the Hepatitis C Virus Glycoprotein E2
Source: mBio. 2017 May 16;8(3):e00382-17. doi: 10.1128/mBio.00382-17 (PMC5433095; doi:10.1128/mBio.00382-17)

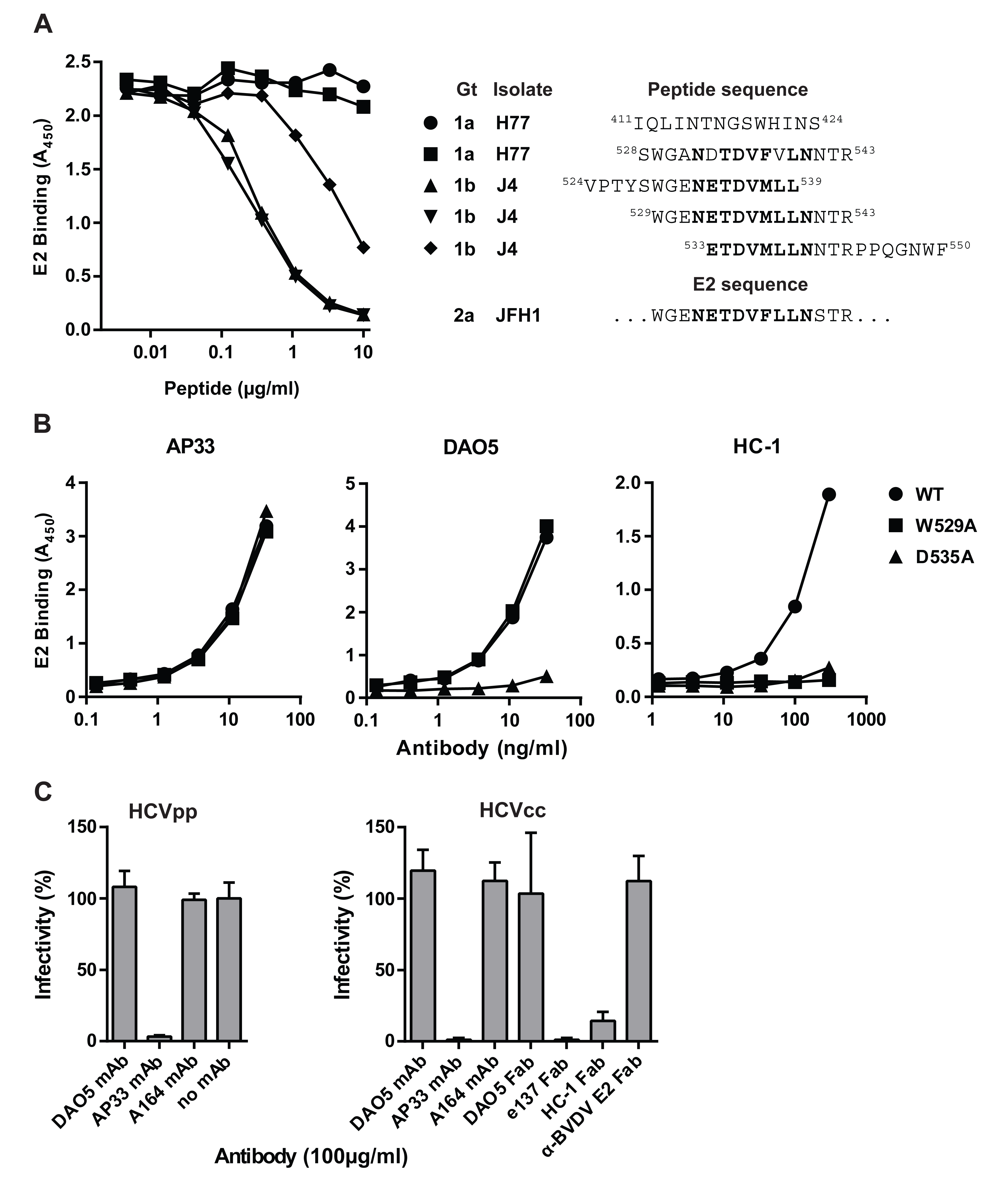

Supplement: FIG S1 [file mbo003173308sf1.tif]

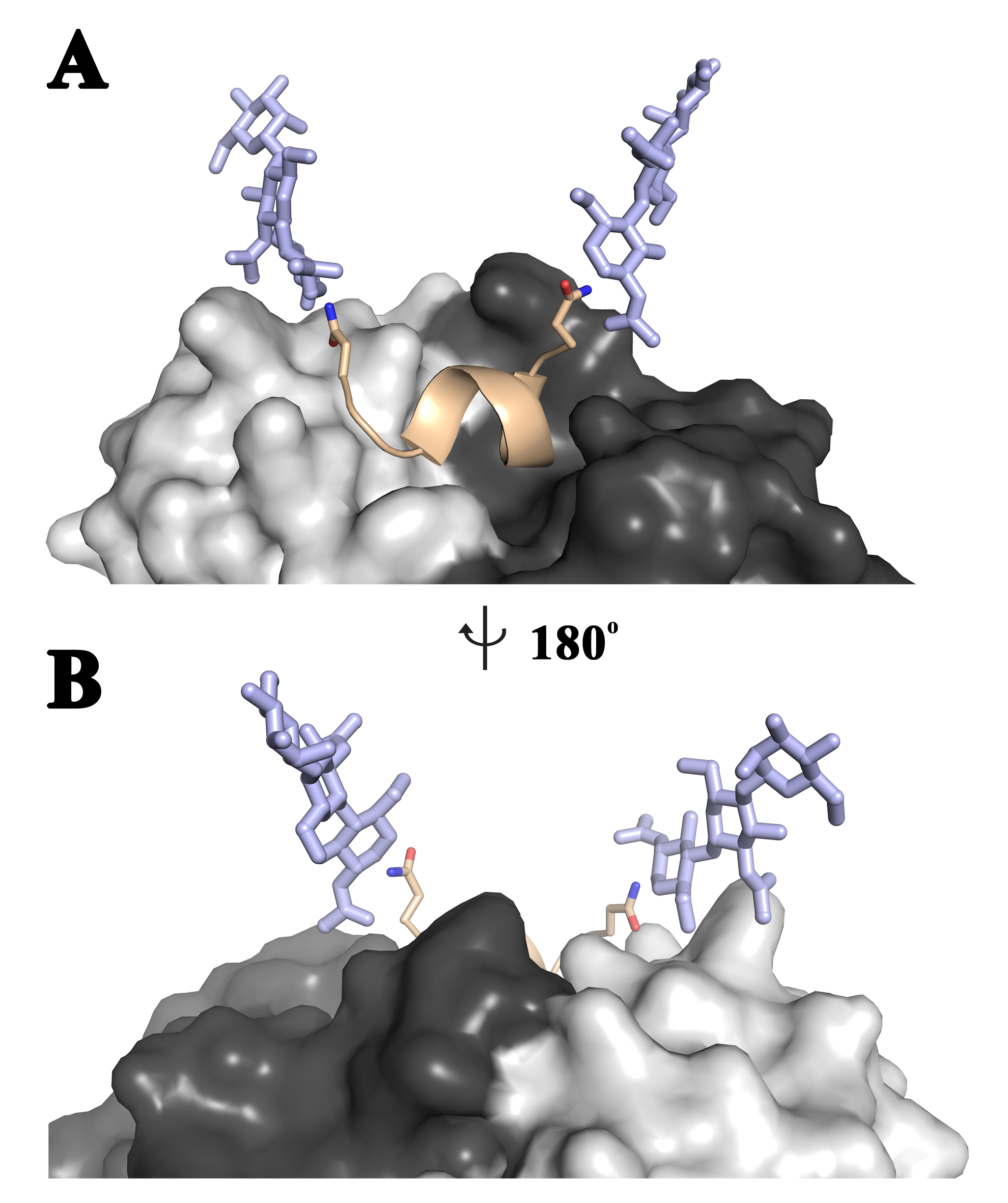

Supplement: FIG S2 [file mbo003173308sf2.tif]
