# Supplementary material for: Conformational Flexibility in the Immunoglobulin-Like Domain of the Hepatitis C Virus Glycoprotein E2
Source: mBio. 2017 May 16;8(3):e00382-17. doi: 10.1128/mBio.00382-17 (PMC5433095; doi:10.1128/mBio.00382-17)
Supplement: TABLE S1 [file mbo003173308st1.docx]

# S1 Table. Diffraction data collection and refinement statistics.

|  | **DAO5 scFv - J4** | **DAO5 scFv - JFH-1** | **DAO5 Fab - J4** |
| --- | --- | --- | --- |
| **Data collection** |  |  |  |
| Space group | *P* 4_1_ 2_1_ 2 | *P* 4_1_ 2_1_ 2 | *P* 2_1_ |
| Protein/ Complexes per AU | 2 | 2 | 1 |
| Cell dimensions |  |  |  |
| *a, b, c* (Å) | 155.94 155.94 61.51 | 155.69 155.69 61.65 | 48.68 80.71 54.57 |
| *α, β, γ* (°) | 90.00 90.00 90.00 | 90.00 90.00 90.00 | 90.00 95.64 90.00 |
| Resolution (Å) | 49.31-2.00 (2.11-2.00) | 49.23-1.90 (2.00-1.90) | 48.44-1.70 (1.79-1.70) |
| Rmerge | 0.107 (0.516) | 0.137 (0.614) | 0.04 (0.228) |
| Completeness (%) | 97.6 (83.5) | 98.5 (99.8) | 97.1 (95.6) |
| Redundancy | 5.9 (2.9) | 4.4 (4.3) | 5.7 (5.7) |
| I/ σ^I^ | 10.3 (1.9) | 9.5 (3.2) | 27.6 (6.8) |
|  |  |  |  |
| **Refinement** |  |  |  |
| No. reflections | 47116 | 58950 | 44787 |
| Rwork/ Rfree | 0.1979 / 0.2176 | 0.1875 / 0.2039 | 0.1774 / 0.2032 |
|  |  |  |  |
| **No. of atoms** |  |  |  |
| Macromolecules | 3727 | 3728 | 3361 |
| Ligand | 0 | 0 | 0 |
| Water | 269 | 326 | 329 |
| Residues per AU | 484 | 485 | 437 |
|  |  |  |  |
| **B-factors** |  |  |  |
| Average B-factor (Å^2^) | 28.67 | 20.84 | 20.02 |
|  |  |  |  |
| **Ramachandran Statistics** |  |  |  |
| favored | 96.6% | 97% | 97.9% |
| allowed | 3.4% | 3% | 2.1% |
| outliers | 0% | 0% | 0% |
|  |  |  |  |
| **R.m.s. deviations** |  |  |  |
| Bond length (Å) | 0.010 | 0.010 | 0.010 |
| Bond angles (°) | 1.08 | 1.06 | 1.12 |

^1^ Values in parentheses correspond to the highest resolution shell. rmsd, root-mean-square deviation.
